# Modeling of the general and primary incidence of borderline mental disorders of the Russian population in the period 1992-2022

**DOI:** 10.1192/j.eurpsy.2026.11305

**Published:** 2026-07-31

**Authors:** V. Mitikhin, T. Solokhina

**Affiliations:** Mental Health Research Centre, Moscow, Russian Federation

## Abstract

**Introduction:**

The *general incidence* of borderline mental disorders (*BMDg*) in the Russian population (per 10,000 population) increased in the period under review from 106.41 to 140.85 (an increase of 32.4%). In 2022, the BMDg index in Russia was 52.7% of all mental disorders, and in the structure of primary *incidence*, respectively, about 81.8%. These facts allow us to briefly characterize modern psychiatry as borderline psychiatry.

**Objectives:**

The construction of mathematical models at the population level to assess the dependence of indicators of the general and primary incidence of BMD in the Russian population on demographic, socio-economic factors and the human resource of psychiatric care in the period 1992-2022.

**Methods:**

When forming linear models of the relationship between factors and indicators of BMD incidence, methods of systematic data analysis and statistical analysis were used. To build a nonlinear logistic model of the general incidence of BMD in the population, the least squares method was used using the MS Excel add-in “Solver”.

**Results:**

The number of human resources (Ps) for the mental health service and society is obviously the main important factor determining the level of the registered patient population. A significant contribution to the change in BMDg value is made by the Po factor (population size) during the period under review. For the primary incidence of BMD (or BMDp), in addition to Ps and Po factors, significant factors are Ey (life expectancy for the Russian population, years) and Un (unemployment rate, %). Linear regression models for BMDg and BMDp indicators with a high level of significance (determination coefficients R^2^ = 0.88 and 0.86, respectively) were obtained. A nonlinear logistic model has been developed to predict the BMDg index when the personnel resource of the psychiatric service (Ps) and the population size (Po) change. Using the logistic model, an estimate of the maximum BMDg index for the Russian population was obtained, which is equal to 19.1% of the Russian population. Since the BMDg index in Russia currently accounts for about 53% of all mental disorders, if this structure is maintained, we will get a value of about 36% of the Russian population for the proportion of all mental disorders. This value practically coincides with the result obtained in the well-known work [1].

**Conclusions:**

The use of operational (using linear models) and long-term (based on a logistic model) estimates of the general and primary incidence of BMD in the Russian population makes it possible to timely monitor, predict and take into account the impact of demographic, socio-economic factors in the life of the population, as well as the human resources of the psychiatric care system. [1]. Wittchen, H. U., Jacobi, F., Rehm, J., et al. *European Neuropsychopharmacology.* 2011; 21: 655-679. doi:10.1016/j.euroneuro.2011.07.018

**Disclosure of Interest:**

None Declared

